# Management of Osteomyelitis in Sickle Cell Disease: Review Article

**DOI:** 10.5435/JAAOSGlobal-D-20-00002

**Published:** 2020-09-02

**Authors:** Humaid Al Farii, Sarah Zhou, Anthony Albers

**Affiliations:** From the Division of Orthopaedic Surgery, McGill University, Montreal, Quebec, Canada.

## Abstract

Sickle cell disease (SCD) is an autosomal recessive disorder that is characterized by abnormal “sickle-shaped” erythrocytes. Because of their shape, these erythrocytes are more likely to become trapped in small slow-flowing vessels, leading to vaso-occlusion. Because this commonly happens in the bones, patients with SCD are at an increased risk for orthopaedic manifestations such as osteomyelitis, septic joint, or osteonecrosis. Osteomyelitis is a serious and potentially disabling condition but can be difficult to differentiate from benign conditions of SCD, such as vaso-occlusive crisis. Diagnosis of osteomyelitis requires careful evaluation of the clinical presentation, laboratory testing, and imaging. Treatment of osteomyelitis in patients with SCD may be medical or surgical, but considerations in antibiotic selection and management preoperatively and postoperatively must be taken to ensure optimal outcomes.

Sickle cell disease (SCD) is the most commonly inherited hematological disorder, affecting millions of patients worldwide.^1^ It is estimated that the overall prevalence of SCD in African-Americans is one in 365.^2^ This condition produces abnormal hemoglobin, which leads to the “sickling” of red blood cells.^1^ Patients frequently present with hemolytic anemia and microvascular occlusion leading to vaso-occlusive crisis (VOC). Patients with SCD are also at an increased risk of orthopaedic manifestations such as osteomyelitis, septic joint, or osteonecrosis.^3^ Osteomyelitis is one of the most serious and potentially disabling orthopaedic manifestations of SCD,^3^ but its differentiation from benign SCD conditions such as VOC can be difficult.^4^ This article aims to discuss the pathophysiology, clinical presentation, diagnosis, and management of osteomyelitis in the SCD population.

## Pathophysiology

**Figure 1:**
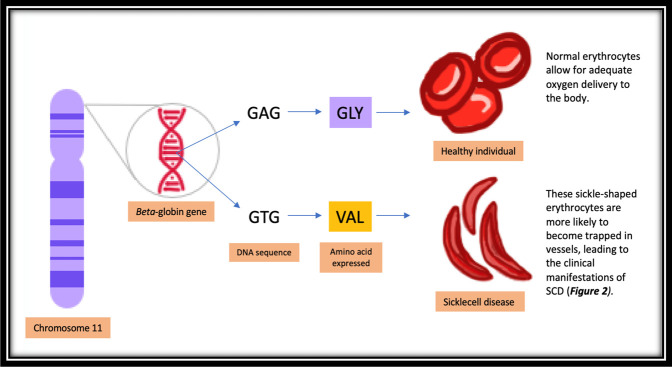
Illustration showing the pathophysiology of sickle cell disease from the level of the chromosome to the morphology of the erythrocyte.

The genetic basis of SCD arises from a point mutation of the beta-globin subunit gene on chromosome 11. This leads to the replacement of the amino acid glutamic acid with the less polar amino acid valine and, consequently, the production of hemoglobin S.^3^ Hemoglobin S has a tendency to polymerize in its deoxygenated state, causing erythrocytes to become crescent-shaped or better known as “sickle”-shaped.^5^ Compared with normal erythrocytes, sickle cell erythrocytes have a markedly reduced lifespan and because of their stiff architecture are more likely become trapped in slow-flowing venules (Figure 1).^5^

Sickle cell trait occurs in heterozygote carriers of this mutation.^6^ Carriers have relative protection against *Plasmodium falciparum* malaria fatality, explaining the high prevalence of the gene in geographic areas of endemic malaria.^6^ However, carriers of the sickle cell trait usually do not develop clinical manifestations of the illness. This review focuses on SCD, which is of autosomal recessive inheritance.

**Figure 2:**
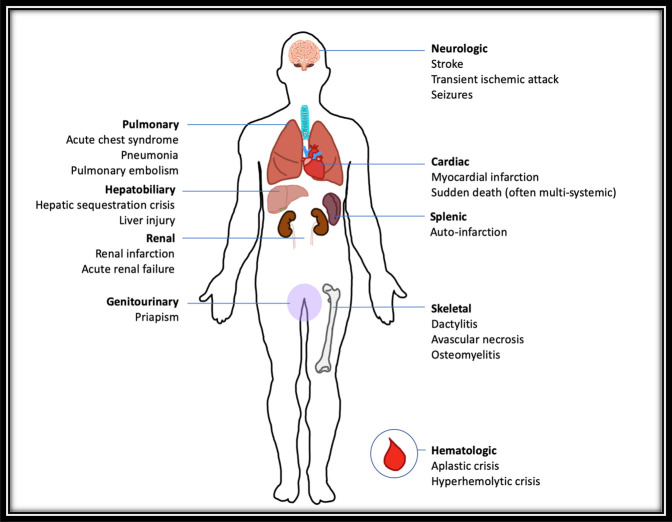
Illustration showing that sickle cell disease (SCD) may present with various clinical manifestations because the disease affects many organ systems.^1^

In SCD, the abnormal erythrocytes can precipitate acute episodic clinical events. The accumulation of sickle cells in the blood stream can occlude the microvasculature, leading to bone infarction and activation of inflammatory pathways.^1^ VOC, also known as sickle cell crisis, can affect multiple systems, including the splenic, hepatic, renal, nervous, and musculoskeletal systems (Figure 2).^1^ Children with SCD develop functional hyposplenia because of autoinfarction, which generally occurs before the age of five.^7^ This impairs the body's immune response against encapsulated organisms.^7^ Combined with the presence of infarcted bone, sickle cell patients are found to be at a greater risk for osteomyelitis.^8^

In the general population, *Staphylococcus aureus* (*S aureus*) is the most common organism associated with osteomyelitis.^9^ In Africa and the Middle East, it is also the most common pathogen of osteomyelitis (39% and 62%, respectively) compared with other pathogens isolated from patients with SCD.^9^ However, in America, Gram-negative enteric bacilli such as *Salmonella spp.* (70%) are more commonly found in the SCD population than *S aureus* (16.4%).^9^ It is postulated that these enteric bacteria arise from ischemic infarction of bowels during vaso-occlusive episodes.^8,10^

## Clinical Manifestations

In the acute stage of presentation, VOC and osteomyelitis are nearly indistinguishable, although VOC is up to 50 times more common than osteomyelitis in patients with SCD.^11^ In both cases, patients may present with fever and a painful, swollen limb with limited range of motion.^12^ Usually, osteomyelitis affects the diaphysis of long bones, such as the femur and humerus, but it is possible for any other bone to be affected.^13^ Berger et al^4^ found that fever and pain identified for at least 24 hours before presentation and swelling of the affected limb were important predictors of osteomyelitis in children with SCD. Their results also suggest that patients with osteomyelitis were less likely to have multiple painful sites than those experiencing a vaso-occlusive episode.

Laboratory testing is not always reliable in distinguishing osteomyelitis from VOC because both conditions can be associated with leukocytosis and elevated inflammatory markers (C-reactive protein and erythrocyte sedimentation rate).^11^ The benchmark in diagnosing osteomyelitis is having a positive culture from either a sample of bone, synovial fluid, or blood.^14^ However, the absence of a positive culture does not rule out the possible diagnosis of osteomyelitis.^8^ Because obtaining bone cultures is extremely invasive and blood cultures are frequently falsely negative,^8^ integrating imaging studies is crucial to identify osteomyelitis in patients with SCD. Timely identification of osteomyelitis is necessary to begin appropriate antibiotic therapy and prevent the need for surgical intervention.

## Diagnosis

OM is difficult to differentiate from VOC in patients with SCD. Although VOC is much more common, OM is an important diagnosis which must not be overlooked. Clinical features, laboratory findings, and radiological features must be used together to guide further diagnostic tests and management. The following modalities can identify radiographic features that may raise clinical suspicion of OM.

## Plain Radiographs

In the early stages of osteomyelitis and VOC, plain radiographs are usually found to be either normal or only showing soft-tissue edema, periostitis, or osteopenia.^3,8^ The lytic changes suggestive of osteomyelitis lag at least 2 weeks behind the process of the infection on radiographs.^15^ Thus, the poor sensitivity and specificity for early detection by plain radiography prompts the need for evaluation with other imaging modalities.^11^

## Ultrasonography

**Figure 3:**
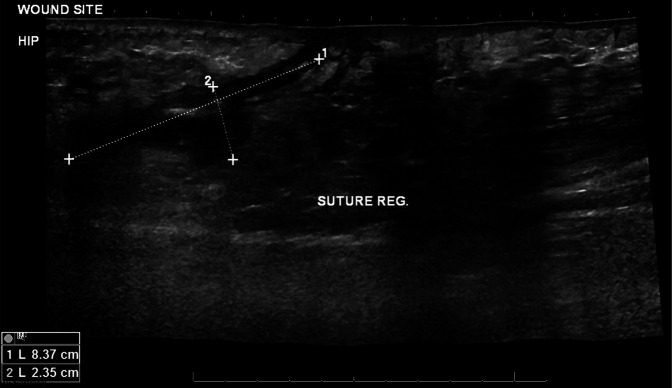
Ultrasonography of the lateral soft-tissue of the hip showing a subcutaneous collection measuring 8.4 cm by 2.4 cm. This collection was used as an indirect indication of the presence of OM because it is likely to have formed secondary to the infection.

Ultrasonography is a rapid and noninvasive investigation that has the ability to show acute findings of extraosseous pathology and/or periosteal elevation in osteomyelitis.^16^ In patients with an elevated C-reactive protein and/or white cell count on admission, the sensitivity of ultrasonography has been shown to be as high as 76% to detect osteomyelitis in SCD.^11^ Although subperiosteal fluid may also be found in VOC, it has been reported that a subperiosteal fluid collection >4 mm is a strong indicator of osteomyelitis (Figure 3).^16^ The mentioned findings are indirect signs of the presence of osteomyelitis but are effective tools when correlated with clinical evidence. With the expansion of ultrasonography as a low-cost and portable device, ultrasonography may be a useful modality for detecting early indirect signs of osteomyelitis in patients with SCD, especially in low-resource settings.

## MRI

**Figure 4:**
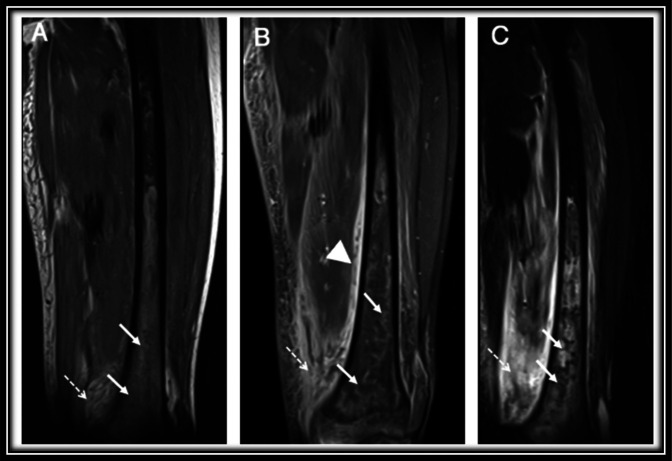
Coronal T1 (**A**), coronal T1 postcontrast (**B**), and coronal short tau inversion-recovery (**C**) images of the femur demonstrating extensive marrow edema with associated serpiginous and tubular marrow enhancement in the distal femoral metaphysis/diaphysis (solid arrows) with extensive associated periosteal reaction (arrowhead), adjacent soft-tissue swelling, and edema (dashed arrows). These findings are characteristic of osteomyelitis. The figure reproduced with permission from Kosaraju et al.^13^

MRI is the imaging modality of choice for diagnosis of osteomyelitis^17^ because the sensitivity has been reported up to 100% (Figure 4).^18^ Early pathological features, such as bone marrow edema, are detectable as early as 24 hours after infection begins.^17^ On MRI, bone marrow edema typically presents as a localized marrow abnormality of decreased signal on T1-weighted images and increased signal on T2-weighted images (Figure 4).^15^ Other secondary findings of osteomyelitis such as soft-tissue collections, cellulitis, and cortical bone sinus tracts also have similar MRI signalling characteristics (decreased on T1, whereas increased on T2).^17^ Used consecutively, MRIs may also be correlated with clinical evidence to evaluate the response to antibiotic therapy, without risk of ionising radiation.

Gadolinium enhancement can improve the accuracy of MRI because the regions of infection show substantial contrast enhancement after gadolinium administration.^3^ Unless contraindicated (ie, impaired renal function), gadolinium contrast imaging is typically performed with MRI to help identify and characterize complications such as abscesses or sinus tracts, which may be missed otherwise.^17^

It is important to correlate the clinical presentation with the MRI findings because MRI is reported to have lower specificity than sensitivity (75% to 96%, vs 82% to 100%).^18^ This is because many of the MRI findings of OM and VOC overlap, and currently no reliable imaging parameter exists to effectively differentiate these diagnoses.^16^ MRI findings may overestimate the severity of the infection or may be indistinguishable from other pathologies such as malignancy.^17^ Finally, although MRI is a powerful diagnostic tool, it remains costly and may not be suitable for young children, patients with metal implants, or situations requiring imaging of large parts of the body.^15^

## Radionuclide Imaging

**Figure 5:**
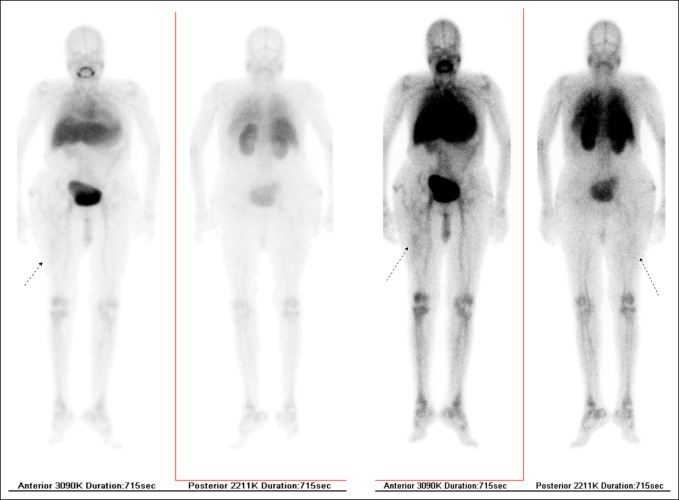
Radiograph of LeukoScan showing increased uptake at right thigh (arrow), which may indicate infective pathology.

Radionuclide scans may be used in situations when the diagnosis of osteomyelitis remains unclear (Figure 5).^15^ Given the high diagnostic accuracy of MRI, it is much less commonly used. A summary of the available scans is provided in Table 1. Because there is a high dose of radiation encountered during nuclear medicine scanning, these scans should be reserved in practice for specific cases in which the benefits outweigh the potential harm.

**Table 1:** Radionuclide Imaging Modalities Available for Detection of Osteomyelitis

Type of Scan (Substance Used)	Function of the Scan	Limitations	Diagnostic Accuracy for Osteomyelitis
Bone scintigraphy (technetium-99m-labelled polyphosphate)	Isotopes first accumulate in capillaries and perivascular space, then in areas of reactive new bone formation.^18^	In settings of impaired blood supply (eg, vaso-occlusion), false negatives may occur at the site of infection.	For acute hematogenous osteomyelitis (95% CI):^9^ Sensitivity 0.81 (0.68-0.90) Specificity 0.84 (0.60-0.97)
Gallium scan (gallium salts, eg, gallium citrate and gallium nitrate)	Increased isotope uptake in areas of inflammation: infection, inflammatory conditions, and malignancy.^18^	The scan does not show bony detail, and it is difficult to differentiate bone from soft-tissue inflammation. The scan also takes 48-72 hours to complete.^18^	For osteomyelitis (unspecified):^18^ Sensitivity ∼60% Specificity ∼80% Combined with bone scintigraphy for vertebral osteomyelitis:^20^ Sensitivity 91% Specificity >90%
Leukocyte imaging (eg, indium-labelled leukocytes, LeukoScan^a^)	Labelled leukocytes are used to localize infection.^18^	Similar limitations to the gallium scan.	LeukoScan:^18^ Sensitivity 90-93% Specificity 85-89%

CI = confidence interval

a LeukoScan is Tc-99 m–labeled antigranulocyte monoclonal antibody Fab' fragments.

Although OM and VOC might be difficult to distinguish based on clinical picture in patients with SCD, a combination of the clinical features, laboratory, and radiological workups all together will help clinicians distinguish one diagnosis from the other (Table 2).

**Table 2:** Clinical Features and Findings Differentiating VOC From Osteomyelitis

Factor	VOC	Osteomyelitis
Prevalence	50× more common than osteomyelitis	—
History and physical examination		
Fever	Fever (>38.0°C) is possible	Fever (>38.0°C) more likely to be identified for 24 hours before presentation
Location of pain	May have multiple sites of pain	More likely to have pain in a single area, usually the diaphysis of a long bone
Joint appearance	Joint swelling is possible	More likely to present with joint swelling
Laboratory testing		
Leukocytes	Normal to mildly elevated	May be elevated
Inflammatory markers (CRP, ESR)	Normal to mildly elevated	Although not specific for osteomyelitis, more prominent elevations in CRP and ESR are found in osteomyelitis
Cultures	—	Benchmark diagnosis is a positive culture from bone, blood or synovial fluid
Imaging		
Plain radiograph	Usually normal, but both conditions may show soft-tissue edema, periostitis, or osteopenia	
MRI	—	Localized marrow abnormality of decreased marrow signal on T1-weighted images and increased signal on T2-weighted images

CRP = C-reactive protein; ESR = erythrocyte sedimentation rate; VOC = vaso-occlusive crisis

## Management

The optimal treatment of osteomyelitis includes a combination of antibiotics with adequate antimicrobial coverage and, if necessary, surgical management through débridement and wound reconstruction. In addition to medical and surgical management, optimization of patients with SCD requires complete patient care including adequate nutrition, smoking cessation, and paying attention to the underlying chronic medical conditions.^15^

### Medical Management

Initial management typically includes IV hydration, oxygenation, and pain control until the diagnosis of OM has been made. Unless the patient shows signs of sepsis and hemodynamic instability, appropriate cultures should be obtained before administrations of antibiotics to increase the likelihood of obtaining a pathogen. Empiric antibiotics can then be discontinued if the diagnosis of OM is ruled out.

In general, the typical duration of parenteral antibiotic treatment of osteomyelitis runs 4 to 6 weeks.^15^ Trials of extended courses of either parenteral or oral antibiotics have not suggested improved outcomes compared with 6 weeks of therapy.^21^ If patients are deemed to be stable enough for discharge, outpatient intravenous antibiotics may be administered through a peripherally inserted central catheter line.

As described previously, *S aureus*, Salmonella, and other gram-negative bacilli are the most commonly isolated organisms from individuals with osteomyelitis in the SCD population. Ideally, the results of a positive blood culture, biopsy, or aspiration should be used to direct the choice of antibiotic. Currently, there is a paucity of literature to direct antibiotic selection for patients with SCD with osteomyelitis, as per the 2016 Cochrane review.^22^ Thus, discussion with an infectious disease specialist to determine the choice of antimicrobial therapy and length of treatment is imperative and performed on a case-by-case basis.^22^ Almeida and Roberts^8^ recommended first-line treatment of confirmed or suspected osteomyelitis to be a third-generation cephalosporin to ensure coverage of the aforementioned organisms. Another reasonable combination for empiric antibiotic treatment is a combination of vancomycin and ciprofloxacin.^23^ Further studies are needed to clarify the most effective antibiotic selection for osteomyelitis in the SCD population. Examples of antibiotic regimes for the common offending bacteria of osteomyelitis in SCD have been provided in Table 3.

**Table 3:** Preferred First-Line Antibiotic Regimens, Based on the Causative Organism

Organism	Preferred first-line regimen
Anaerobes	Clindamycin 600 mg IV q6h, or Ticarcillin/clavulanate, 3.1 g IV q4h
Enterobacteriaceae, quinolone-sensitive	Ciprofloxacin 400 mg IV q8-12h
Enterobacteriaceae, quinolone-resistant	Piperacillin/tazobactam, 3.375 g IV q6h, or Ticarcillin Clavulanate, 3.1 g IV q4h
*Pseudomonas aeruginosa*	Cefepime, 2 g IV q8-12h, with ciprofloxacin 400 mg IV q8-12h
*S aureus*, methicillin-sensitive	Cefazolin, 1-1.5 g IV q6h, or Nafcillin or Oxacillin, 1-2 g IV q4h
*S aureus*, methicillin-resistant	Vancomycin 1g IV q12h, or if allergic, Linezolid 600 mg IV q12h
*Streptococcus* species	Penicillin G, 2-4 million units IV q4h

Adapted from Hatzenbuehler et al (2011).^21,24,25^ Adaptations are themselves works protected by copyright. So in order to publish this adaptation, authorization must be obtained both from the owner of the copyright in the original work and from the owner of copyright in the translation or adaptation.

### Surgical Management

As stated above, MRI can be useful for evaluating the response of osteomyelitis to antibiotic treatment. Remaining or nonresponsive nidi of infection, or areas of necrotic bone, require surgical débridement, followed by antimicrobial therapy.^23^ Evaluation of the patient and their most recent MRI by an orthopaedic surgeon is essential for optimal outcomes. In compromised hosts, surgical management may not be feasible in all cases. If the host is severely compromised and the risk of operation outweighs the benefits, the option for treatment may only be nonsurgical (such as antibiotic suppression). In extreme cases, amputation may be necessary as a lifesaving intervention.^15^

Surgical treatment of osteomyelitis includes surgical débridement to remove necrotic and infected material and is followed by soft-tissue coverage using either direct closure or flap coverage. Intraoperative cultures are typically sent to confirm the offending pathogen. If a patient commenced empiric antibiotic therapy and is no longer acutely ill (ie, no evidence of soft-tissue infection or sepsis), it is optimal for antibiotics to be discontinued for at least two weeks before débridement. This allows for greater accuracy in microbiologic identification.^15^

Débridement of bone is performed until punctate bleeding is found, also known as the “paprika sign.”^26^ In immunocompromised patients such as the SCD population, margins of at least 5 mm are recommended to reduce the risk of recurrence.^15^ Within the area of débridement, local antimicrobials may be placed directly to the site of infection via absorbable (ie, calcium sulfate beads) or nonabsorbable carriers (ie, polymethyl methacrylate).^27^ These are used to sterilize the area and temporarily maintain the dead space but eventually can be replaced after 2 to 4 weeks with cancellous bone graft.^15^ During the process of healing, skeletal stability may need to be increased to reduce stress on the affected bony area and surrounding soft tissues. In general, external fixation is recommended because internal fixation has a tendency to become secondarily infected.^15^

After débridement, adequate soft-tissue coverage of the bone may be done via either direct closure or flap coverage.^15^ Direct closure is preferred when surgical intervention results in a minimal defect, in which the bone and soft tissues can be enclosed easily. However, with large soft-tissue defects or when there may be exposed bone, joint, or tendons, local muscle flaps or a free flap can improve the healing environment by improving vascularization. This brings natural host defense mechanisms and also improves antibiotic delivery.^15^ When flap coverage is necessary, consultation with plastic surgery is suggested for optimal management. Complete wound closure is always recommended because healing by secondary intention may cause avascular scar tissue to develop in the defect.^15^

**Figure 6:**
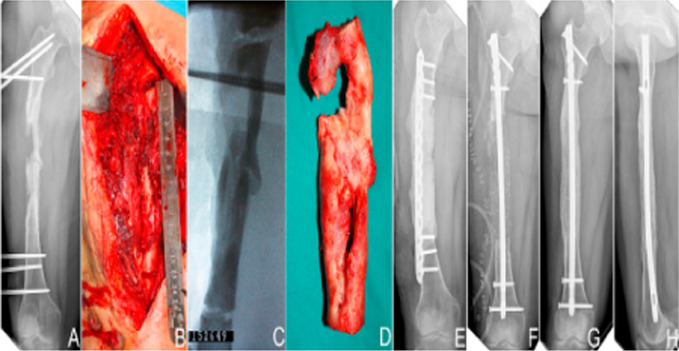
**A**, Preoperative AP radiograph of posttraumatic femoral infection demonstrating notable bone loss secondary to débridement in a 30-year-old female patient. **B** and **C**, Segmental resection of infected bone (aggressive surgical débridement) according to intraoperative fluoroscopy location. **D**, Excision of infected bone. **E**, First stage postoperative AP radiograph demonstrated that bone defect was filled with antibiotic bone cement spacer, and locking compression plate was used to limb stabilization after débridement. **F**, Second stage postoperative AP radiograph demonstrating placement of iliac crest autograft after removal of bone cement and replacement of locking compression plate with intramedullary nail to limb stabilization. **G** and **H**, AP and lateral radiographs showing bony consolidation 15 months after bone grafting. The figure reproduced with permission from Han et al.^30^

More recently, the induced membrane (Masquelet) technique has been introduced for the management of bone defects after débridement of osteomyelitis.^28,29^ First, a cement spacer is placed into the defect. The spacer is made of polymethyl methylacrylate, which serves to prevent scar tissue from developing in the defect, and inducing osteogenesis through releasing osteoprogenitor cells. A second operation is performed 6 to 8 weeks later to carefully incise the induced membrane and replace the spacer with autologous bone graft (Figure 6).^28^ Giannoudis et al^28^ reported harvesting this graft material from the femoral intramedullary cavity with a reamer/iIrrigator/aspirator (RIA) device. Because it is a relatively novel technique, further studies are necessary to determine the efficacy of the Masquelet technique in achieving bone union and eradication of infection.

A systematic review by Morelli et al^29^ found that almost 50% of patients had complications (superficial or deep surgical site infections) and that 18% of patients required reintervention (because of persistence of infection or nonunion).

## Perioperative Considerations

Orthopaedic intervention in the SCD population carries a higher risk of complications in comparison with patients without SCD undergoing similar procedures.^31^

Preoperatively, evaluation of patients for transfusion includes serial hemoglobin, hemoglobin S level (HbS%), renal function, liver function, and oxygen saturation.^32^ Simple transfusion therapy (also known as conservative transfusion) is used to increase hemoglobin levels to 10 g/dL using sickle-negative blood.^31^ This has been shown to provide similar outcomes to aggressive transfusion, which targets to decrease HbS% to <30%.^31^ Exchange transfusion is another preoperative transfusion technique, which involves the removal the patient's sickled blood and replacing it with donor packed red blood cells. Exchange transfusion has been found to have similar outcomes as simple transfusion but requires a larger amount of blood to be transfused and results in a higher rate of transfusion-related complications.^33^

With general anesthesia, patients with SCD have an increased risk for acute chest syndrome (ACS) and VOC.^32^ ACS is a life-threatening condition secondary to occlusion of the pulmonary vasculature, which is defined as fever and respiratory symptoms with newfound pulmonary infiltrates found on chest radiograph.^1,8^ Initial therapy requires fluid management, supplementary oxygen, pain management, bronchodilators, and empiric antibiotics.^32^ In cases of worsening hypoxemia despite supplemental oxygen, urgent transfusion therapy is warranted.^1^ Perioperatively, patients with SCD should have careful monitoring of cardiac rhythm, blood pressure, and oxygen saturation.^34^ Because they are also at an increased risk for hypothermia, intraoperative warming measures (ie, warming blanket) are also of benefit.^34^

**Figure 7:**
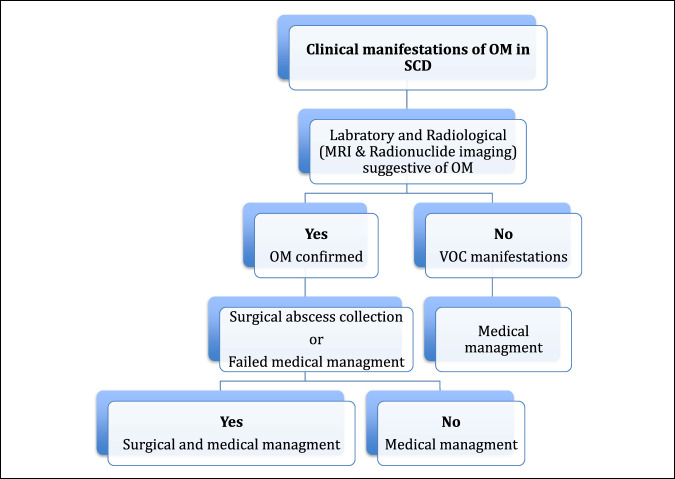
Preferred algorithm by authors for management of OM in sickle cell disease (SCD).

After surgery, patients with SCD are encouraged to use incentive spirometry and chest physiotherapy to prevent ACS.^31^ Appropriate IV antibiotics are also given, particularly based on the sensitivities of the organisms of the infection. Finally, supplemental oxygen and IV hydration are imperative to prevent the occurrence of vaso-occlusive crisis.^31^ Resuming regularly prescribed medications, such as hydroxyurea or L-glutamine, are to be considered at this time as well. Yawn et al^35^ recommended that during hospitalization, individuals with SCD should continue on their usual dose of hydroxyurea, unless they are found to have developed secondary cytopenia or are pregnant/breastfeeding. We (as authors) preferred certain algorithm that illustrated in the Figure 7 to manage osteomyelitis in SCD.

## Summary

Osteomyelitis is serious medical condition that can be difficult to identify, especially in patients with SCD. Although VOC is a much more common presentation, clinicians must consider the possibility of osteomyelitis when evaluating patients with SCD who present with fever and a swollen limb. Advancements in imaging such as MRI and radionuclide scanning may be helpful in differentiating the two conditions. If infection remains present despite antibiotic therapy, adequate surgical management is necessary to ensure optimal outcomes.
